# On the Reparative Process in the Human Tendons after Subcutaneous Division for the Cure of Deformities

**Published:** 1861-05

**Authors:** 


ART. VI.—*On the Reparative Process in the Human Tendons after Subcutaneous Division for the Cure of Deformities; with an account of the appearances presented in fifteen post-mortem examinations in the human subject; also a series of experiments on rabbits, and a resumé of the English and foreign literature of the subject.* Illustrated by Seven Lithograph Plates and a Series of Wood-cuts. By WILLIAM ADAMS, F.R.C.S., Surgeon to the Royal Orthopedic and Great Northern Hospitals; Lecturer on Surgery at the Grosvenor Place School of Medicine; late Demonstrator of Morbid Anatomy at St. Thomas's Hospital. Churchill: London, 1860. pp. 175.

WE are under obligations to the author of this work for a personal compliment. It is not necessary, however, that a feeling of friendship should interfere to make men praise him; his labors have already sufficiently achieved that object, and we are merely serving mankind in the regular discharge of our duty.

Mr. Adams believes he has established the following propositions:—

"1. That tendon is one of the few structures of the body, such as bone, cellular tissue, nerve tissue, and blood-vessels, capable of reproduction or regeneration; and that the newly formed tissue acquires, within a few months of its formation, the structural characters of the old tendon, so perfectly as to be, under the microscope, with difficulty distinguishable from it; but it does not acquire, (at least it has not up to three years, the latest period to which these observations extend,) through its substance, the uniformly opaque, pearly lustre of old tendon. In the mass it retains a grayish, translucent appearance, streaked only with opaque fibres at a late period, so that the recent section affords an easy method of distinguishing the new from the old tendon.

"2. That when a tendon has been divided subcutaneously, for the cure of club-foot, and its cut extremities are separated and held apart during the active period of the reparative process, *i.e.* the first two or three weeks, as by mechanical extension employed with variable rapidity in different cases, according to the activity of the reparative process, new tendon is formed of variable length, according to the extent of the separation, for the purpose of reuniting the divided extremities of the old tendon."

The greatest length he has seen in a human being of a newly-interposed piece, perfectly formed, and of thickness equal to that of the divided cord, was two inches and a quarter. This was in a girl aged nine years. It is probable that, in children, the length is generally from half an inch to an inch, and in adults, from one to two inches.

"3. That the process—is essentially similar in animals and in man. That the perfection of the process is in exact proportion to the absence of extravasated blood and inflammatory exudation; and that the sheath of the tendon, when consisting of loose-textured areolar tissue, as in the tendo Achillis, and other tendons surrounded by soft tissues, is of importance: 1, in preserving a connection between the divided extremities of the tendon; 2, in furnishing the matrix in which the nucleated blastematous or proper reparative material is effused; and, 3, in giving definition and form to the newly developed tendinous tissue.

"4. That the ultimate perfection of the reparative process by the regeneration of tendinous structure, and the elongation of a shortened muscle by the insertion of a portion of new tendon into its length, equal in strength to the old tendon, and closely resembling it in its microscopic characters, is marred only by the adhesion of the deep surface of the new tendon, to a greater or less extent, with the neighboring fibro-cellular tissue. These adhesions may limit the free play of the tendon, but will not interfere with sufficient motion being obtained. In cases of relapsed deformity, however, in which the operation of tenotomy may be repeated, these adhesions will, in many cases, prevent sufficient separation of the divided extremities of the tendon being obtained. Therefore, if much separation be required, a second operation is generally unsatisfactory in its result; and beyond a second operation, very little advantage can ever be obtained from operative treatment. Hence the necessity of the closest attention to the treatment after the first operation, and the explanation of the complete failure after repeated operations.

"5. That the perfection of the reparative process, especially in non congenital cases—in which more or less paralysis frequently exists, may be interfered with by injudicious after-treatment, especially by the too early and too rapid mechanical extension. And—that when tendons situated in dense fibrous sheaths, of a tubular form, are divided, there is great danger of complete non-union; the divided extremities of the tendon becoming adherent to the inner surface of the sheath without any direct connection with each other,—and, when the operation is performed near to such portions of the sheath, the extension must be conducted very slowly.

"6. That there is no reason for believing that—direct approximation and re-union of the divided extremities of the tendon must be first obtained, and—the required elongation—afterwards—be procured by gradual mechanical extension of the new connecting medium—occupying at least a month or six weeks; according to—some orthopedic authorities of the present time; but—that the required length of new tendon should be obtained during the time occupied in its formation, *i.e.* from about two to three weeks under the ordinary conditions of health; but in paralytic cases, and—patients of feeble health, this period may be doubled. Therefore, the object of gradual mechanical extension during this time, is to regulate the length of new tendon: 1, by forcibly overcoming ligamentous resistance in some cases—and, 2, by preventing a too rapid and extensive separation of the extremities of the tendon, in other cases, especially in those of non-congenital origin, in which many of the ligaments offer no resistance, and the reparative power is so feeble, from partial or complete paralysis, that rapid extension, separating too widely the divided extremities, would endanger their union, and also when tendons situated in or near dense, tubular sheaths—are divided.

"7. That the new tendon remains during life as a permanent tissue, and as an integral portion of the tendon the divided extremities of which it has been formed to reunite."

He takes some pains to justify his disbelief that the new matter progressively contracts till it forms only a "linear cicatrix," the muscular matter lengthening to an equivalent extent.

"8. That the effect of this permanent elongation—is not only to correct deformities mechanically—but that—its higher physiological or dynamic object is to allow of motion being gained—voluntary motion is also obtained, and is followed by an increased development of the muscular structure.

"9. That when recontraction of the foot takes place, and the deformity returns at a distant period after tenotomy, this does not depend upon absorption of the new material—but upon structural alterations in the muscular tissue."

These propositions, which we have abridged, are a summary of the conclusions principally advanced and supported in this very beautiful book. It can certainly not be denied that Mr. Adams's volume has the aspect and attributes of sterling merit. He gives us many dissections, some from his friends, a copious series of experiments on animals, seven fine plates, and a sheet of wood-cuts, and, what has not been always justly or fully done among the Anglo-Saxons, a historical sketch of the progress of the discovery and invention of tenotomy. Still, however, as he very honorably states himself, "even at the present day, opinions differ very widely both with respect to the actual phenomena observed during the reparative process, and also as to the general pathology of the reparative process in tendons after division." Several of the propositions enumerated above are contradicted by the very highest authority, and we cannot compliment him as having settled the whole theory and practice. Perhaps, soon, some critic of industry, candor, and large practical experience, and who has exhausted the experimental part of this inquiry, some man who has no temptation to signalize himself by "peculiar views," may arise to show that, except in condemnation of each other, the competing authors are all right; that the study is extensive and diversified; and that every theory and each practice which has been advanced by such honorable men, will find its proper place in the complete survey of the whole. Our limits will not permit us to go much further than to state some of the discrepancies to which we allude.

His first proposition, as cited above, cannot, we believe, be controverted, unless, as we shall see, in assuming the identity of the new matter with tendon.

No. 2 has, we think, the prevailing authority in its favor; yet the school which he opposes with so much earnestness, and which believes that the new matter contracts into the *linear cicatrix* he alludes to, opposes this, and contains some certainly authoritative names. He enumerates Tamplin, Broadhurst, and Holmes Coote; and his quotation from Stromeyer must be considered as disagreeing with him.

A number of his quotations deny the truly tendinous character of the newly formed cord, allowing it not to contract into a linear cicatrix. He himself enumerates Pirigoff, Von Ammon, and Dieffenbach; and Paget, in his quotation, styles it exceedingly rare.

The whole memoir leaves upon our mind a strong feeling of suspicion that the general contractive tendency of all newly-formed connective membrane, which has explained such curious results in the morbid anatomy of the kidneys and liver, must shorten the new tendon, and hence prove one of the causes of ill success.

To No. 3 we presume no exception is to be taken, unless a microscopic one. The exclusive agency of "nucleated blastema," in the regeneration of tendon, seems to us hardly a thing to be conceded. Several authorities allege the active mediation of cells; and even Mr. Adams acknowledges that some of them are present.

Our only objection to No. 4 is one which we have cited above, the tendency to contraction of all connective cicatrixes.

At No. 5 we would express a doubt of the expediency of operating on many of the feeble and paralytic cases. On the impropriety of incising tendons in the vicinity of dense fibrous sheaths, and still more, of synovial capsules, the arguments of Mr. Adams appear to us conclusive and important.

No. 6 is perhaps plausible; but, in the present state of opinion among operators, it seems to us that one can hardly consider it settled. Is it not a problem to be decided by accumulated and general experience?

On No. 7 we have already said all that we imagine necessary.

No. 8 we judge as sound as possible.

To No. 9 we can hardly consent to subscribe. The facts alluded to have a woeful inclination toward the theory of contraction of cicatrixes in connective membrane.

We confess that we do not very clearly comprehend, as just, the supposition ascribed to the honored name of Stromeyer, that division of its tendon debilitates a muscle by depriving it of its stimulant to contraction, so weakening it as to permit its ultimate elongation. We have always fancied that when a muscle was allowed to contract without antagonism, the weakness which it acquired was connected with shortening, and that, though its contractile power might decay, its capacity of being lengthened would be diminished. As far as we can understand the phenomena thus explained, we should judge them to be owing to other causes.

